# Product News

**DOI:** 10.1155/S1463924683000413

**Published:** 1983


Journal of Automatic Chemistry, Volume 5, Number 3 (July–September 1983), pages 160–167

### Product News

### 34th Pittsburgh Conference and Exposition

The Pittsburgh Conference on Analytical Chemistry and Applied Spectroscopy continued its record of successful meetings with the 34th Pittsburgh Conference and Exposition held in Atlantic City, New Jersey from 7 to 12 March 1983. Total attendance showed a 9·3% increase over the previous year. The organizers recently released some comparative statistics:

| | 1983 | 1982 |
|---|---|---|
| Conferees | 14 720 | 12 387 |
| Exhibitors | 7008 | 7497 |
| Total Registration | 21 728 | 19 884 |
| Exhibiting Companies | 579 | 560 |
| Booth Spaces | 1475 | 1373 |
| Seminar Rooms | 34 | 35 |
| Technical Papers | 948 | 849 |

The following new products are some of those exhibited at the conference. (The 35th Conference, again in Atlantic City, is to be held from 5 to 10 March 1984.)

### Pittsburgh: IBM Instruments Computer System

To date (June 1983) marketed only in the domestic United States, the IBM Instruments Computer System can increase laboratory productivity by providing instrument control, data acquisition, and data-analysis capabilities. Graphic presentation of results can be obtained on a CRT display and/or on hard copy. Results can also be communicated to other systems.

An easy to use, real-time, multi-tasking operating system combined with specially designed data systems hardware allows users to perform many functions:

- Operate several instruments
- Monitor instrument progress
- Collect, store, and analyse data from instruments under program control
- Build or modify procedures
- Print or plot results of completed work
- Store or transmit files for later use
- Process data.

The system has been designed for maximum flexibility for current requirements with the ability to expand for future needs. The modular design permits the mixing and matching of various features to meet specific requirements: this allows users to tailor a system that may range from a low-priced configuration dedicated to a single instrument, to a fully programmable large configuration supporting many instruments and additional laboratory data-processing. The optional BASIC programming language can be used to extend the programmed capabilities of the system. All of the system's features are designed for quick installation.

The system was developed to meet the analytical needs of research, development, quality control, testing and clinical laboratories.

*A brochure is available from Gary L. Gisle, Director of Marketing and Services, IBM Instruments Inc., Orchard Park, PO Box 332, Danbury, Connecticut 06810, USA. Tel.: 203 796 2500.*

**Circle No. 2 on Reader Enquiry Card**

### Pittsburgh: Analytical Products Division, Sybron Corporation

The newly formed Analytical Products Division of Sybron demonstrated their Photo-chem total organic carbon analysers and Servomex oxygen analysers.

The 'Persulffate' injector for the Photo-chem uses photochemical (UV) oxidation combined with a highly sensitive resistivity detection system to provide total organic carbon determination free of inorganic carbon interference. It operates in a range of 20 000 ppm to 50 ppb; the instrument can analyse anything from high-purity process water to industrial effluent. The Servomex oxygen analysers (for biomedical, medical and physiological research, and applications in chemical processing and combustion-efficiency monitoring) use a permagnetic principle of measurement. The 1100A, released at Pittsburgh, is microprocessor controlled.

*More information from Sybron Corporation, Boston, Massachusetts 02132, USA.*

**Circle No. 3 on Reader Enquiry Card**

### Pittsburgh: Apples for process control

The Cyborg Corporation launched their ISAAC/Apple data acquisition and control system at the Conference. It can accept up to 512 direct inputs from sensors; sources may include any mix of thermocouples, RTDs, strain-gauges, pressure transducers, flow meters, or any analogue or digital signal. The system can be configured as a standard data logger or industrial controller, but offers the added benefit of the Apple II. Suggested applications are: process monitoring, data logging, pilot plant operation, small-scale process control and industrial R&D.

Among the advantages of the ISAAC/Apple system are that all sources can be directly connected to the system's screw-type terminals, and modular inputs can be located next to the primary sensors up to 250 ft from the computer.

*Details from Cyborg Corporation, Newton, Massachusetts 02158, USA.*

Circle No. 4 on Reader Enquiry Card

### Pittsburgh: Fluorometer

The Nova is a microprocessor-based ratio-recording spectrofluorometer made by Baird Corporation. The machine includes as standard many features which are optional extras in its competitors. Nova's operating parameters are selected on a keyboard and continuously displayed on a built-in CRT monitor—in digital or bar graph format. Peak height or peak area may be selected for optimizing integration modes. Features include holographic gratings, selectable excitation and emission slit widths, and excitation, emission and synchronous scanning capability.

*Details from Baird Corporation, Bedford, Massachusetts 01730, USA.*

Circle No. 5 on Reader Enquiry Card

### Pittsburgh: Dual Sample autoinjector/Dual variable wavelength detector

A new version of the 725 AutoInjector is able to derivatize and inject samples in a single step; up to 32 samples can be analysed unattended. The unit—the *Model 725D*—is being promoted to laboratories where routine rapid pre-column derivatization of sample is possible. It was initially developed for amino-acid determinations in the brewing industry and is expected to be useful for HPLC applications in biotechnology, pharmaceutical, food and beverage industries (the Dual Sample AutoInjector used with a simple HPLC system may replace analysers costing up to three times more). The chromatographer simply loads alternate vials with sample and reagent into a quadrant (loading the necessary number of vials and quadrants). Up to four quadrants can then be inserted onto the standard 725 carousel; derivatization and LC injection is then only a matter of pressing a button. The 725D can also be used for injecting large sample volumes—to 1460 $\mu$l.

Micromeritics' *788 Dual Variable Wavelength Detector* was also displayed at the Conference. It uses two user-selectable UV/Vis wavelengths to analyse chemical compounds of like or very similar molecular structure. Chromatographers can use the 788 to resolve overlapping peaks without changing analysis conditions or resorting to expensive detection methods. Monitoring of a sample at two different wavelengths allows sample constituent comparisons based on absorbance ratio, absorbance sums, absorbance differences. Results reflect real comparisons because detection occurs in a single sample cell.

The 788 can be used to verify the purity of eluting compounds, selectively eliminate unwanted peaks, collect pure sample components, and double characterize chemical species.

*Further information from Micromeritics Instrument Corporation, 5680 Goshen Springs Road, Norcross, Georgia 30093, USA. Tel.: 404 448 8282.*

Circle No. 6 on Reader Enquiry Card

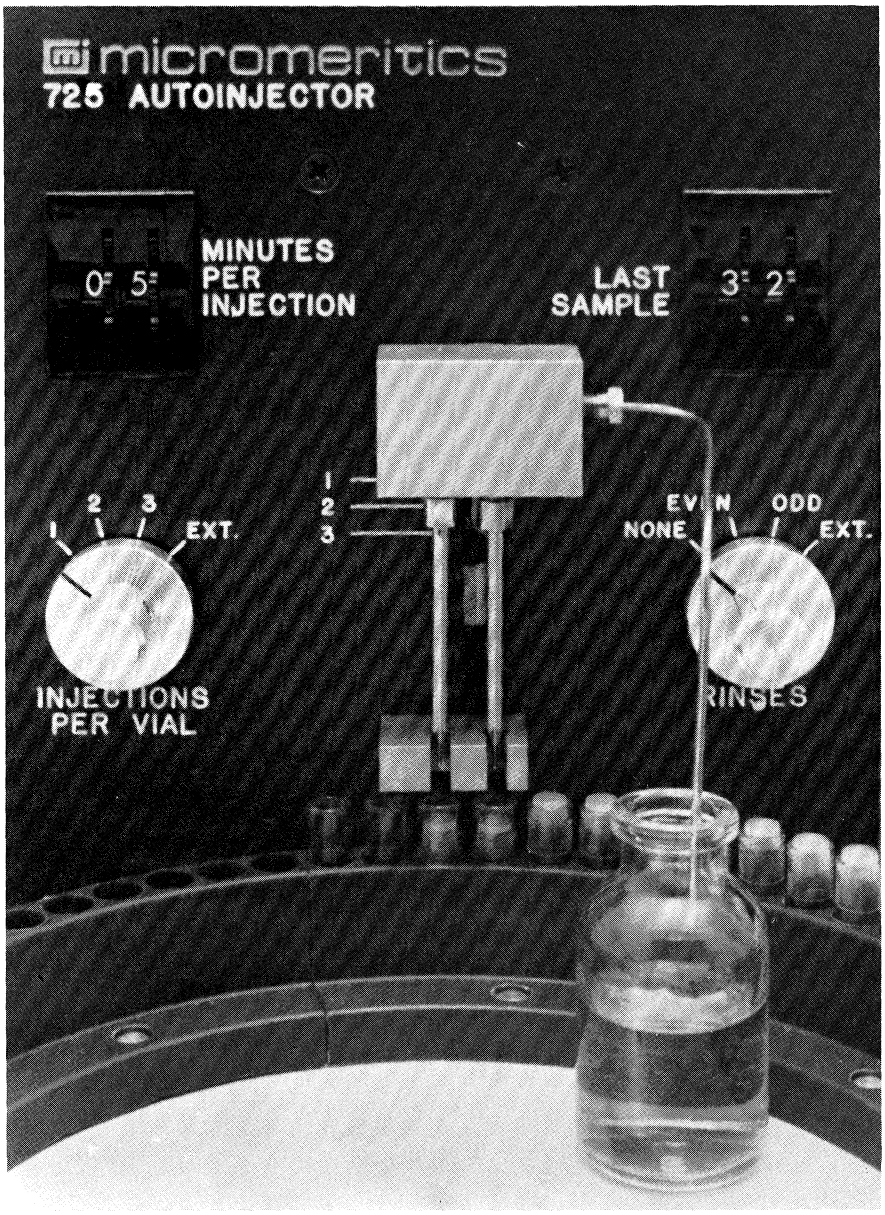

*Those familiar with the original Model 725 will appreciate the 725D's adherence to the basic design principles that have made the 725 popular for automating routine liquid chromatograph injections. The photograph illustrates the only noticeable feature difference: the dual positive sample displacement mechanism and special mixing tee.*

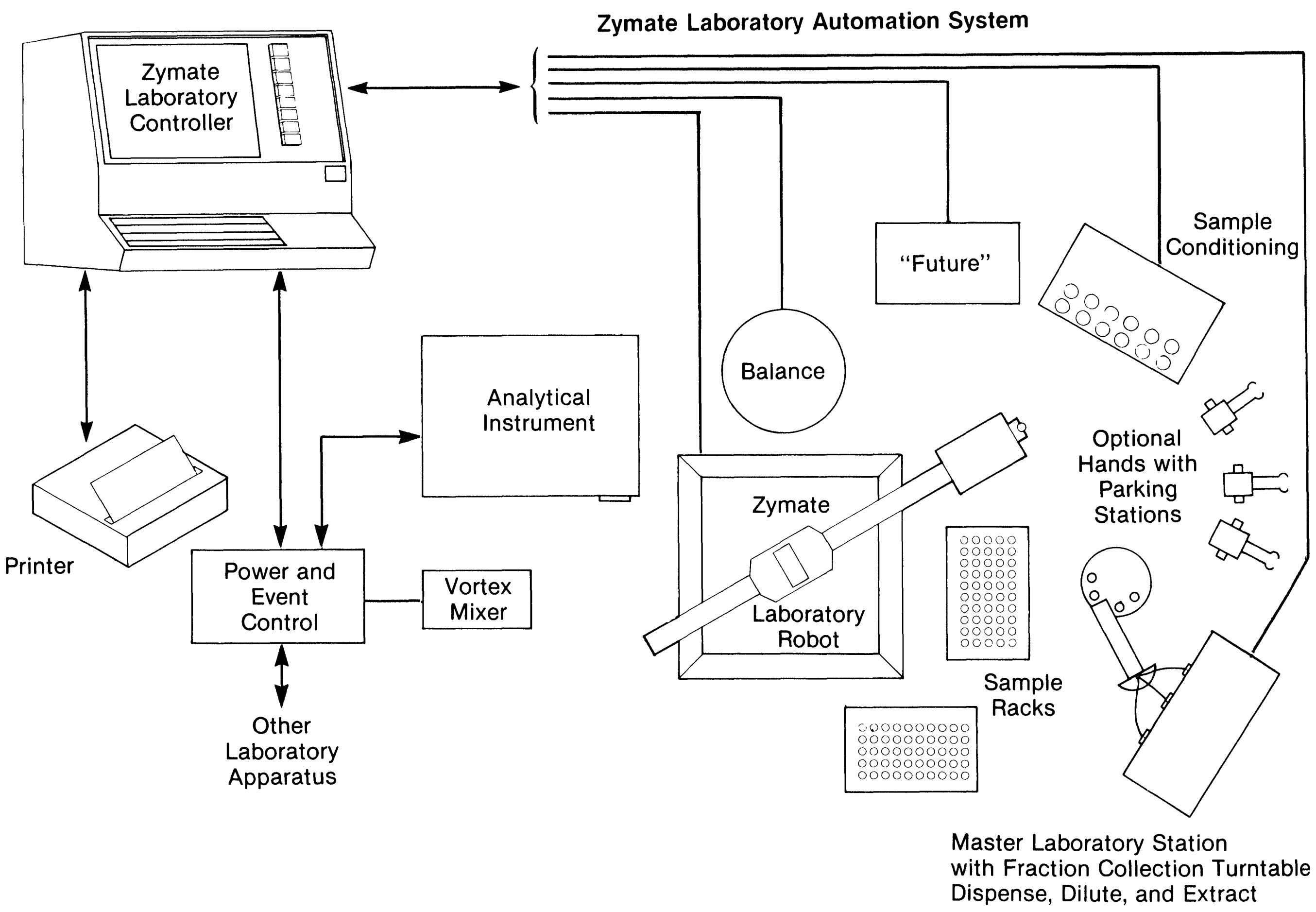

*The Zymate Laboratory Automation System: typical applications include automated liquid-handling, filtration, extraction, partition, chemical derivatization, sample conditioning and sample introduction. (Zymark Corporation, Hopkinton, Massachusetts, USA.)*

### Pittsburgh: Sample preparation

The microprocessor-controlled Zymate laboratory automation system is described, for those who did not get to Atlantic City, in a 12-page booklet. The Zymate combines robotics and laboratory stations to automate sample-preparation procedures; the diagram shows the system relationship between the robot, stations and controller.

The laboratory robot transfers samples from station to station according to user-programmed procedures. When the sample preparation procedure is completed, the robotic arm either introduces samples directly into the analytical instrument or places them in a carousel or rack for subsequent analysis. Since the robotic arm is capable of automatically changing hands, several hands may be used in the procedure.

The laboratory controller uses microprocessor technology to control the robot and laboratory stations and to communicate with the operator. In essence, it manages the sample-preparation procedure in terms of the laboratory unit operations, their sequence and timing. Controller programming is menu-based, with tables and prompts similar to commercial word-processors. Sequential steps can be initiated using familiar laboratory terminology. Sample preparation methods can be stored for future recall by using the floppy-disc drive.

Laboratory stations, placed within reach of the robotic arm, are electronically connected to the controller. As samples are brought to each station, the controller sets all the necessary operating parameters. For example: when dispensing, the robot moves a test-tube or vial to the Master Laboratory Station (MLS) dispenser outlet. The controller then instructs the MLS to dispense the programmed amount of the specified reagent or solvent. The controller waits for a 'completed' signal from the MLS and, then, instructs the robot to move the sample to the next operation.

Zymark, the manufacturer, say that the Zymate gives quality analytical results, increased productivity, improved methods development, convenience and safety, and the possibility of future expansion.

*The booklet about Zymate (a newsletter called 'Laboratory Automation for Chemistry and Biochemistry' is published by Zymark and worth requesting at the same time) is available from Zymark Corporation, Zymark Center, Hopkinton, Massachusetts 01748, USA. Tel.: 617 435 9041.*

Circle No. 7 on Reader Enquiry Card

### Pittsburgh: HPLC detector/autosampler for AA, flame, HPLC, UV/Vis

ISCO, Inc. (Lincoln, Nebraska) announced several new products including the ISIS auto-sampler and the ISCO $V^4$ variable HPLC detector.

The ISIS (at $2495; $3195 with wash station and microprocessor sequencer; $4995 for the complete autoinjector system) is easy to interface with a spectrometer or any other analyser using liquid samples. The accessory autoinjector includes all the components to automate any liquid chromatograph.

The company list eight reasons for buying their $V^4$ variable HPLC detector:

(1) Over 10 times normal deuterium lamp life.
(2) Quick warm-up.
(3) Lowest priced variable detector [at Pittsburgh].
(4) Choice of built-in 10 cm chart recorder or LED read-out.
(5) Three-year warranty.
(6) Optional peak selector.
(7) Ten different cells for LC, HPLC, and micro HPLC fit slip-out cassette. Volumes as low as 0·4 $\mu$l.
(8) 190–750 nm range, 6 nm bandwidth, 0·002 AUFS sensitivity, $2 \times 10^{-5}$ A noise.

*Details from ISCO, Inc., Box 5347, Lincoln, Nebraska 68505, USA. Tel.: 800 228 4250.*

**Circle No. 8 on Reader Enquiry Card**

### Pittsburgh: Continuous-flow analysis

A system incorporating a computer and interface designed to collect, analyse and correct data from segmented-flow continuous analysis wet-chemical systems was displayed by Scientific Instruments Corporation. In selecting peak responses the CFA-85 system uses a combination of peak detection and time windows, which allows the data system to account for abnormal responses. The user can apply three corrections, if necessary, to results: base-line, sensitivity and carry-over alterations. The CFA-85 is recommended for TOC and TOD analysis of water samples, electrolytes in blood, or librium and valium assay.

*Further information from Scientific Instruments Corporation, 25 Broadway, Box 295, Pleasantville, New York 10570, USA.*

**Circle No. 9 on Reader Enquiry Card**

### Pittsburgh: Rapid scanning high-resolution spectrometer

Spectra Span VI, from Beckman Instruments, combines a computer-controlled echelle scanning monochromator with a direct-current plasma (DCP) source to determine elemental components in liquids and gases by plasma emission. It features easy-to-use software, rapid scanning, cursor-prompted instructions on screen, and a unique wavelength drive. The Spectra Span analyses samples with high-total dissolved solids and performs both quantitative and qualitative measurements. Accessories include an auto-sampler, Microsoft BASIC software, a *de luxe* printer (also a standard printer), computer-selected dual photomultiplier detection, and a work-station.

*Further information from Beckman Instruments, Inc., Fullerton, California 92634, USA.*

**Circle No. 10 on Reader Enquiry Card**

### Pittsburgh: Liquid chromatograph

The Model 5500 was launched by Varian Associates at the Conference; it features fully-integrated, intelligent control; microstep pumping; programmable detection with scanning; microbore capability; structured upgradable software; and complete stand-alone automation.

All system components—injectors, pump, detectors, heaters, external events and serial communications—are integrated and share resources in a single compact unit. An interactive CRT/keyboard controls and simplifies instrument operation. The CRT presents 'real-time' status displays of all chromatographic conditions for the system and for each component. The user can build or modify any method at any time; and, while the LC 5500 is running one method, the CRT can be used to build another without interference. The LC 5500 uses the same pump as the existing 5000 series LC systems, and delivers the microflow rates required for new microbore chromatography, as well as the flows needed for normal and semi-preparative separations.

The column/detector compartment is designed to accommodate a broad range of built-in detectors (up to two at a time) including the new UV 200 UV-Vis detector with time programmable wavelengths and scanning. This detector features a patented flow-cell that maximizes light throughput and minimizes the RI effects, providing the performance needed for both microbore and conventional HPLC.

For automatic, unattended operation, an auto-sampler can be added and methods set up in the 5500 will control automatic injection of up to 60 samples. For complete automation, including data handling, the Model 5500 interfaces with the VISTA 402 Chromatography Data System or with an external computer.

*For more information contact Varian Associates Ltd, 24–28 Manor Road, Walton-onThames, Surrey UK, or Varian AG, Steinhauserstrasse, CH 6300 Zug, Switzerland.*

**Circle No. 11 on Reader Enquiry Card**

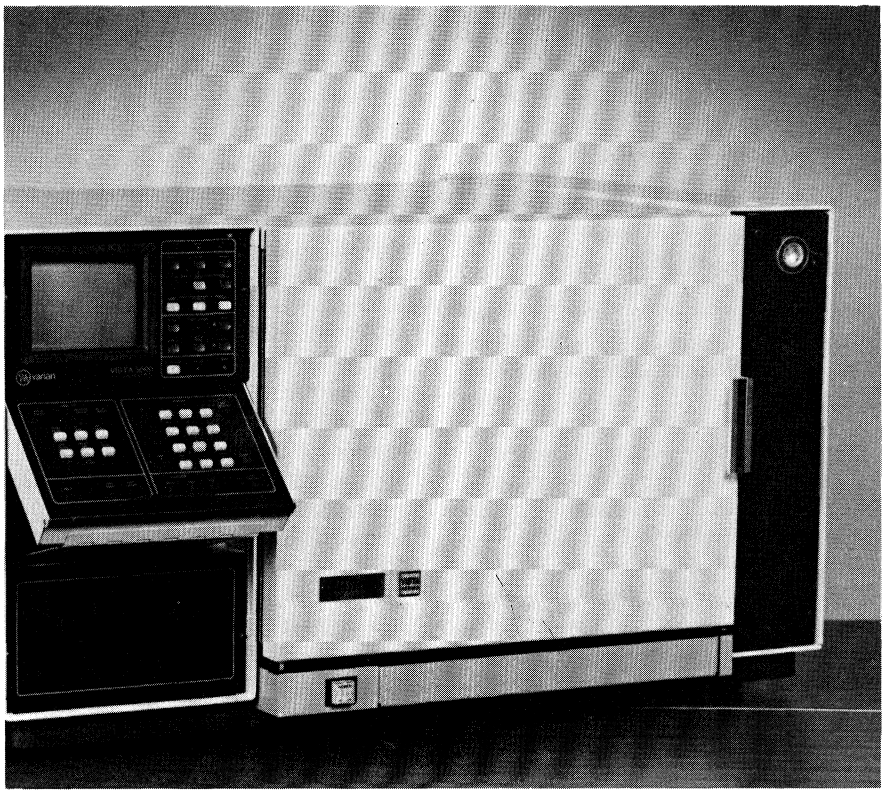

*The Model 5500 liquid chromatograph. Modular construction allows the user to choose the best combination of injectors, heaters, columns and accessories for specific applications. (Varian, Switzerland and UK.)*

### Precious chemicals

A free catalogue of precious metal chemicals is available from Engelhard Industries Ltd. The chemicals listed are normally held in stock by Engelhard or can be supplied at short notice. Each chemical is described in terms of its common name, its IUPAC name, its Engelhard Code number and its formula. An analytical specification is given for each compound and any hazards associated with the material are detailed in a hazard rating guide.

*The catalogue can be obtained from the Publicity Department, St. Nicholas House, St. Nicholas Road, Sutton, Surrey SM1 1EN, UK. Enquiries are also welcomed for other precious metal chemicals not listed or for listed chemicals under different specifications.*

Circle No. 12 on Reader Enquiry Card

### Ion analysis in food and drink

Dionex have announced that the 2000i ion chromatrographs are being used by a number of food processors. It appears that as well as having applications for quality-control procedures, ion chromatography is useful for detecting adulteration and for competitive analysis. Dionex ion chromatography determines ions and ionizable compounds in foods and beverages: the technique is a form of liquid chromatography which uses ion exchange as the mode of separation. Suppressed conductivity detection is used, as well as electrochemical and ultraviolet/visible detection. Analysable compounds include anions ($Cl^-$, $Br^-$, $NO_3^-$, $NO_2^-$, $PO_4^{3-}$, $SO_4^{2-}$ etc.), cations ($K^+$, $Na^+$, $Ca^{2+}$, $Mg^{2+}$ etc.), organic acids, transition metals ($Fe^{2+}$, $Cu^{2+}$, $Zn^{2+}$ etc.), amines, phenols, and surfactants.

*Information on the 2000i ion chromatographs from Dionex (UK) Ltd, First Floor, The Parade, Frimley, Camberley, Surrey GU16 5H7, UK, tel.: 0276 29771; or from the Dionex Corporation, 1228 Titan Way, Sunnyvale, California 94086, USA, tel.: 408 737 0700.*

Circle No. 13 on Reader Enquiry Card

### FPLC system for high-performance chromatography of biomolecules

Pharmacia's FPLC (fast protein liquid chromatography) system was designed for high resolution, high yields, short separation times and for freedom from contamination. The system is not, according to Pharmacia, based on existing HPLC methodologies, rather it is a new approach to high-efficiency chromatography of labile biomolecules. The FPLC system is centred on a series of separation media for ion-exchange chromatography and chromatofocusing, based on a macroporous matrix in the form of monodisperse spheres. These media are complemented by silica-based media for anion-exchange chromatography, with a sample load of 50 mg or more of protein per run. Typical separation times are 10 to 30 min.

Pharmacia have produced a high-precision pump, a gradient programmer and a full range of ancilliary equipment.

*The system is described in a colour booklet available from Pharmacia (Great Britain) Ltd, Prince Regent Road, Hounslow, Middlesex TW3 1NE, UK, tel.: 01-572 7321; or Pharmacia Fine Chemicals AB, Box 175, S-75104 Uppsala 1, Sweden.*

Circle No. 14 on Reader Enquiry Card

### High-vacuum technology

A free 12-page booklet from Javac Ltd of Farnham, UK, covers the terms and formulae relevant to high-vacuum technology. It describes the two main types of mechanical rotary vacuum pumps, direct-drive and belt-driven, and describes how they work and gives the advantages of each. The booklet also contains formulae and charts which will guide the reader to calculate the size of pump for a given application.

*Copies from Javac Ltd, 3 Waverley Lane, Farnham, Surrey GU9 8BB, UK. Tel.: 0252 721539.*

Circle No. 15 on Reader Enquiry Card

### RI detector for liquid chromatography

Refractive index detectors tend to be highly sensitive to slight changes in temperature and also to lamp energy fluctuations—both contribute towards a high noise level.

The first of these problems has been overcome in the new RID-2A from Shimadzu by the large heat capacity of the detector housing and its insulation, eliminating any need for a water-jacket; and by a solid metal inner-casing with a special heat filter next to the lamp housing. The second problem has been solved with electronic circuitry which compensates for lamp energy fluctuation. Finally, the inclusion of double deflection, which amplifies very small refractive index changes, makes the Shimadzu RID-2A a reliable and sensitive instrument.

*The Shimadzu refractive index detector is sold in the UK by Dyson Instruments Ltd from whom further details are available: Sunderland House, Station Road, Hetton, Houghton-le-Spring, Tyne & Wear DH5 0AT, UK. Tel.: 0783 260452.*

Circle No. 16 on Reader Enquiry Card

### SCIC journal

The *Wescan Ion Analyzer* is a newsletter about single-column ion chromatography (SCIC)—a technique that applies high-performance liquid chromatography (HPLC) technology to the analysis of dissolved ions. Published three times a year by Wescan Instruments Inc., the newsletter includes technical notes on new developments in SCIC, applications of SCIC to specific analytical problems, and advice for users on methods optimization and system troubleshooting.

*Free subscriptions are available from Wescan Instruments Inc., 3018 Scott Blvd., Santa Clara, California 95050, USA. Tel.: 408 727 3519. Articles on SCIC for publication in 'Wescan Ion Analyzer' may be submitted to: Thomas Jupille (Editor: 'Wescan Ion Analyzer'), c/o Wescan Instruments, 3018 Scott Blvd., Santa Clara, California 95050, USA.*

Circle No. 17 on Reader Enquiry Card

### Mill for rapid sample preparation

A redesigned and improved Cyclotec Sample Mill has been announced. This new mill has a very low noise level (less than 75 dBA in normal operation) and is equipped with a very reliable transmission. Maintenance demand is low. The Cyclotec is intended for rapid and uniform grinding of a wide variety of feeds, grains, leaves etc. and also for grinding of chemicals, pharmaceuticals and similar products. Features include dust-free operation, no clean-out between samples and no thermal degradation of samples.

*Contact Teactor Ltd, Cooper Road, Thornbury, Bristol BS12 2UP, UK. Tel.: 0454 417798.*

Circle No. 18 on Reader Enquiry Card

### Intelligent printer for process instrumentation

New from Microtrol is an intelligent printer designed for interfacing with instrumentation such as that used in process-control installations. The MED-IQ combines a standard, medium-speed matrix printer with a microprocessor-based control system. This incorporates serial or parallel I/O facilities to RS 232 or Centronics etc. standards, and has the ability to accept analogue inputs. The MED-IQ prints on a 7×5 dot matrix format at a speed of 80 characters/s, and includes a system time clock. Under normal operating conditions, the printer interrogates its associated instrumentation and automatically stores the resultant data; this is then either periodically printed-out at pre-determined time intervals, or on request by the operator.

MED-IQ can be adapted to meet a user's specific print requirements. Up to six different print formats may be defined and manual controls set into the printer's control panel make provision for the operator to print-out the present time or any of the defined formats. Typical print formats include conventional data-logging, for a number of input channels; and logging with time data and embedded text, so that a time-related record of events may be compiled.

*More information from Microtrol Engineering Design Ltd, 640 Melton Road, Thurmaston, Leicester LE4 8BB, UK.*

**Circle No. 19 on Reader Enquiry Card**

### 'Instrumental Thin-Layer Chromatography'

Published in January, TL-10-E is CAMAG-AG's latest catalogue. Descriptions of instruments are accompanied by methodological explanations—so *Instrumental Thin-Layer Chromatography* is a useful guide to current TLC practice.

*The 56-page, coloured brochure is available, free, from Ch. Gfeller, CAMAG-AG, Sonnenmattstrasse 11, CH 4132 Muttenz, Switzerland. Tel.: 061 61 34 34.*

**Circle No. 20 on Reader Enquiry Card**

### Atomic Spectroscopy Data Management Software

ASDMS is a software package that provides data collection, report generation and instrument control and which can be used with Perkin-Elmer's Models 5000, 4000, 2380 and 2280 atomic absorption spectrophotometers interfaced to the 3600 data station. The analyst can use ASDMS to select all the information required for the analysis from the standard methods 'cookbook' stored on disc. Analytical methods developed by the user can also be stored on disc for rapid access. Analytical data from flame, graphite furnace or hydride analyses can be collected in real time and stored, and once all the data is on file, reports can be generated in any of eight different formats. For example, all lead analyses from a batch of samples can be reported separately. Alternatively, each element analysed in a sample can be reported under a single sample number, even though the elements may have been determined by a mixture of methods, for example flame and furnace. Results can also be corrected for weight and dilution.

Instrument control is possible when ASDMS is linked to the Model 5000 or 4000 spectrophotometers. The operating parameters for each element are stored in the methods library and can be used to set up the instrument automatically. Up to six element methods can be grouped and transferred to the instrument.

Perkin-Elmer's HGA graphics software allows high-speed data acquisition: necessary when fast, transient peaks need to be recorded. The peaks can be displayed and compared, allowing the analyst to develop the best method for graphite furnace analysis.

*Further information from Perkin-Elmer Ltd, Post Office Lane, Beaconsfield, Buckinghamshire HP9 1QA, UK. Tel.: 04946 6161.*

**Circle No. 21 on Reader Enquiry Card**

### Clinical chemistry guidelines

The NCCLS (National Committee for Clinical Laboratory Standards) recently published sets of guidelines for laboratories.

*NRSCC1-T* describes the development of definitive methods—a definitive method is an analytical method which has been subjected to in-depth investigation and evaluation for sources in inaccuracy. The Council for the National Reference System in Clinical Chemistry (NRSCC) will be using the guidelines to review 'proposed candidate definitive methods' for submission to the NCCLS consensus process. When a definitive method for a specific analyte has been approved according to the criteria in *NRSCC1-T*, it will be used for establishing the accuracy of the reference method for that analyte.

The other two documents concern calibration and control materials. *C22-T* describes specifications for materials used for the calibration of routine analytical systems for the measurements of analytes in body fluid specimens. The calibration materials referred to in *C22-T* are those that are carried through the same analytical process as the patient specimens. And *C23-T* contains specifications for materials used to monitor routine systems for the measurement of analytes in serums and urine. The guidelines address control materials with an assigned value or range of values and those without an assigned value. Both types may be used to estimate precision, and the former may also be used to estimate accuracy or bias.

*Each document costs $9·00 (plus $1·00 for out-of-US orders) and the NCCLS would like payment with order. The Committee is based at 771 E. Lancaster Avenue, Villanova, Pennsylvania 19085, USA.*

**Circle No. 22 on Reader Enquiry Card**

### Radioactivity meter

Described as a convenient means for verifying radioisotope activities the PW 4600, an intelligent microprocessor-based Microcurie (Becquerel) Meter, is being promoted to nuclear medicine laboratories and to hospitals. The meter consists of a slim microprocessor control console linked to an ionization chamber into which vials or syringes are inserted for checking. 39 different isotopes can be selected for checking using a simple keyboard, which provides a single-key identification of the seven most commonly used labelled materials. Results appear on a digital display; a hard-copy record is produced simultaneously on the unit's built-in strip printer. The results are automatically corrected for isotope half-lives and the print-out includes the date and time of testing. When a sample volume is entered on the keyboard, the meter calculates activity per millilitre; it can also indicate the volume of solution necessary to constitute a predetermined dose. The PW 4660 can be factory pre-set to measure in either millicuries or megabecquerels, and this can be changed subsequently at any time if required.

*Details from Pye Unicam Ltd, York Street. Cambridge CB1 2PX, UK. Tel.: 0223 358866.*

**Circle No. 23 on Reader Enquiry Card**

### Aurora: clinical chemistry analyser

The Aurora computer-controlled clinical analysis system, which can handle 300 tests/h and which allows dialogue (in English, French or German) with the operator, is to be distributed in Belgium, France, Italy, Spain and FR Germany by Behringwerke AG. Behringwerke AG is a subsidiary of Hoechst and it is now entering the routine clinical chemistry market with products for enzyme and substrate determination.

*Details from Aurora's manufacturer: Ultrolab AB, Box 20032, S-161 20 Bromma, Sweden.*

**Circle No. 24 on Reader Enquiry Card**

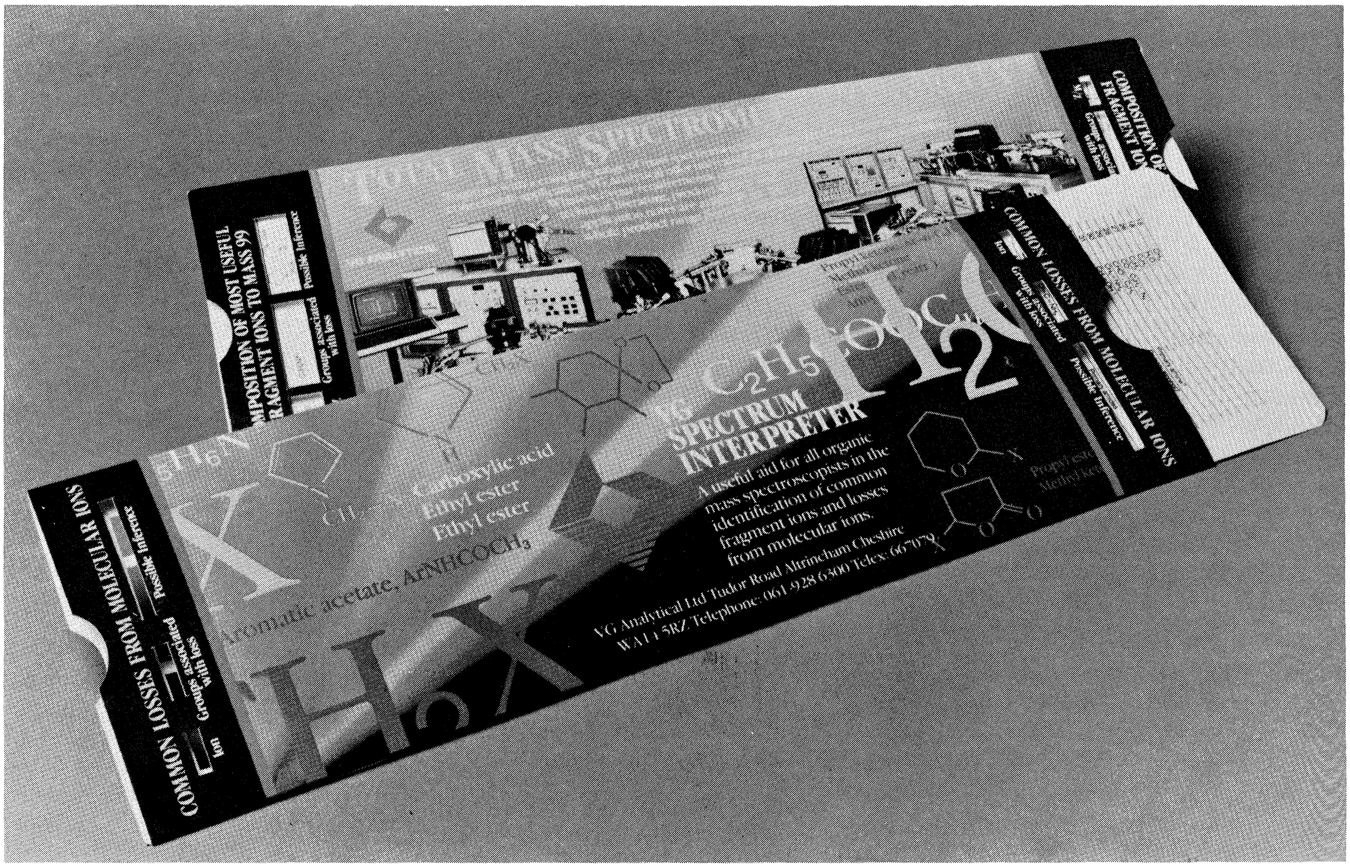

*A free spectrum interpreter is offered by the Cheshire-based manufacturer of mass spectrometers and associated data systems: VG Analytical. The interpreter's slide-rule facilitates the easy interpretation of common losses from molecular ions and shows the composition of fragment ions to Mass 99. Copies from the Marketing Department, VG Analytical Ltd, Tudor Road, Altrincham, Cheshire WA14 5RZ, UK. Tel.: 061 928 6300.*

Circle No. 25 on Reader Enquiry Card

### DIY PCBs

Applied Photophysics offer a series of low-cost, wired and fully tested PCB modules, which should be of interest to those building their own electronic processing and control instruments. By adding a simple power-supply, switches, potentiometers etc. and housing to a PCB module, it is possible to construct a high-performance instrument at a fraction of the cost of a made-up version. All modules are supplied with a full circuit diagram and application notes.

The PCB modules available include a stepping motor control unit designed to drive a reversible motor at speeds adjustable over many decades and a ramp generator board producing positive or negative going ramps covering a $\pm 10$ V range.

The phase sensitive detector module covers the range from 0·01 Hz to 1 MHz, has low noise and a sensitivity of $10\,\mu$V/V. Related modules include a phase control unit and phase-to-voltage converter.

The ratiometer module generates three simultaneous analogue outputs based on the ratio of two input voltages. This module is particularly useful in optical detection schemes where light-source intensities are subject to fluctuations and compensation is required to obtain steady readings. The gated integrator module is a fast, low-leakage, integrator having an input isolating gate which can be opened, under external control, for periods ranging from DC down to 30 ns.

Various programmable time delay modules are offered; all have three outputs, each of which generates a time-delayed pulse following a trigger pulse or transition applied to a single input. Delays are available from 1 ns to 100 s.

The dual wideband amplifier has two identical amplifiers which are independently powered. With a rise time of 35 ns, an input impedance of 1 MΩ and an output impedance of 50 Ω, this amplifier is ideal for many laboratory and industrial applications.

*Information from Applied Photophysics Ltd, 20 Albemarle Street, London W1X 3HA. Tel.: 01 493 4194.*

Circle No. 26 on Reader Enquiry Card

### FIA

A joint venture between Advanced Medical Supplies of Aldershot, UK and the Laboratory of the Government Chemist (LGC) has resulted in a new low-cost flow-injection analysis (FIA) system.

The LGC has developed its own FIA systems for water, food and beer analysis. The LGC and Advanced Medical Supplies analyser (they have been working together over the last few months) will be manufactured and marketed exclusively by Advanced Medical Supplies.

The system is similar to those used by LGC in its water analysis section and is capable of performing a colorimetric examination of water samples. Standard analytical methods include ammonia, nitrate, chloride, orthophosphate, anionic surfactant, inorganic sulphate, iron, free chlorine, pH by electrode, pH by titration and conductivity.

*Further information from Jeff Young, Advanced Medical Supplies Ltd, 19 Holder Road, North Lane Industrial Estate, Aldershot, Hampshire, UK.*

Circle No. 27 on Reader Enquiry Card

### SAM

SAM is a 'synthesis automation machine' for the automated synthesis of polypeptides and oligonucleotide fragments required in recombinant DNA research. Using BIOSEARCH's phosphite-triester synthesis program and BIOSEARCH precursors, reagents, and purification protocols, SAM produces 95% coupling efficiencies, 98% purity of products, and 100 unit (after purification) of a nucleotide 15-mer. Operation of SAM is under the control of a microprocessor, incorporating a 500-step memory capable of handling up to 24 outputs and eight inputs. The machine is preprogrammed to perform oligonucleotide synthesis by the phosphite-triester method with a 21 min cycle time. Capacity for nine additional user-defined programs is also included.

After 20 min of set-up by a technician the machine, unattended, will synthesize a nucleotide 15-mer in 6 h. Three steps are required to execute a synthesis:

(1) Fill the reagent reservoirs with appropriate materials.
(2) Load the reusable reaction column with the requisite derivatized support.
(3) Enter the desired oligomer sequence into the microprocessor memory.

On completion of the synthesis, the support is removed from the column, and the oligomer deprotected and detached from the support. The product is then purified.

SAM can also be used for many nucleotide synthesis methodologies, including phosphate-triester and phosphate-triester chemistries. It is easily programmed for the preparation of oligonucleotides with either mixed base sites or modified base insertions. And it will automate polypeptide synthesis. BIOSEARCH offers a number of components designed to expand SAM's capabilities. By adding supplementary valves, the machine can control additional reagent reservoirs. An attached printer will produce hard-copy verification of program parameters, oligomer sequence entered, and synthetic steps executed. To allow collection of detritylation effluent for colorimetric assay, an effluent diversion valve can be installed.

*Details from New Brunswick Scientific Ltd, 23–34 Emerald Street, London WC1N 3QA, Tel.: 01 404 4515.*

Circle No. 28 on Reader Enquiry Card

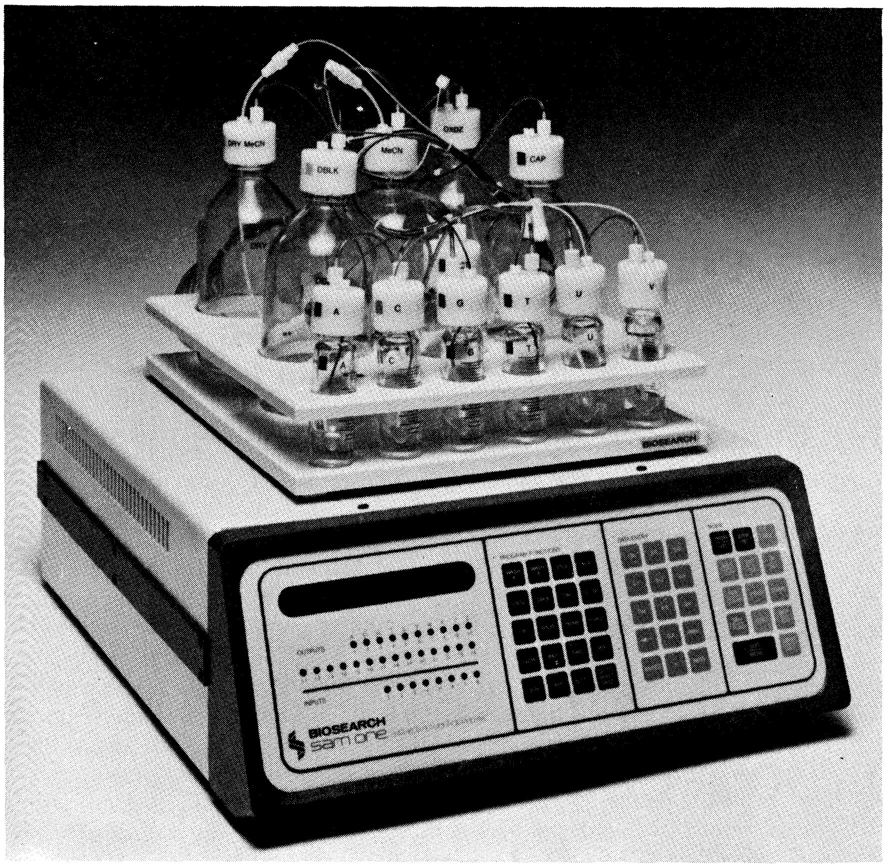

*Costing much less than competing oligonucleotide synthesizers, New Brunswick Scientific's SAM gives its user the flexibility to select the most economical route to a desired synthetic product.*

### 'Differential Scanning Calorimetry'

This is a 44-page booklet offered free of charge by Perkin-Elmer Ltd. It covers the theory, practice and applications of differential scanning calorimetry.

*Copies from Perkin-Elmer Ltd, Post Office Lane, Beaconsfield, Buckinghamshire HP9 1QA, UK. Tel.: 04946 6161.*

Circle No. 29 on Reader Enquiry Card

### 1984 Meetings

**35th Pittsburgh Conference and Exposition**

To be held from 5 to 10 March 1984 in Atlantic City, New Jersey, USA.

*Further information from Mrs Linda Briggs, Pittsburgh Conference, Department J-212, 437 Donald Road, Pittsburgh, Pennsylvania 15325, USA.*

**XIIth International Congress of Clinical Chemistry**

To be held from 29 April to 4 May 1984 in Rio de Janeiro, Brazil.

*Further information from the Executive Secretary, XIIth International Congress, Rua Vicente Licinio 95, Cep 20270, Rio de Janeiro, Brazil.*

**2nd International Congress on Automation and New Technology in the Clinical Laboratory**

To be held from 15 to 18 October 1984 in Barcelona, Spain.

*For further information contact 2nd International Congress on Automation and New Technology in the Clinical Laboratory, IV Congreso Nacional de la Sociedad Española de Quimica Clinica, Apartado de Correos 543, Barcelona, Spain.*

**14th Annual Symposium on the Analytical Chemistry of Pollutants and the 3rd International Congress on Analytical Techniques in Environmental Chemistry**

To be held from 22–24 November 1984 at the Palacio de Congresos in Barcelona, Spain.

*For further information contact 14th Annual Symposium—3rd International Congress—Expoquimia, Avda. Reina Mª. Christina, Barcelona 4, Spain.*

